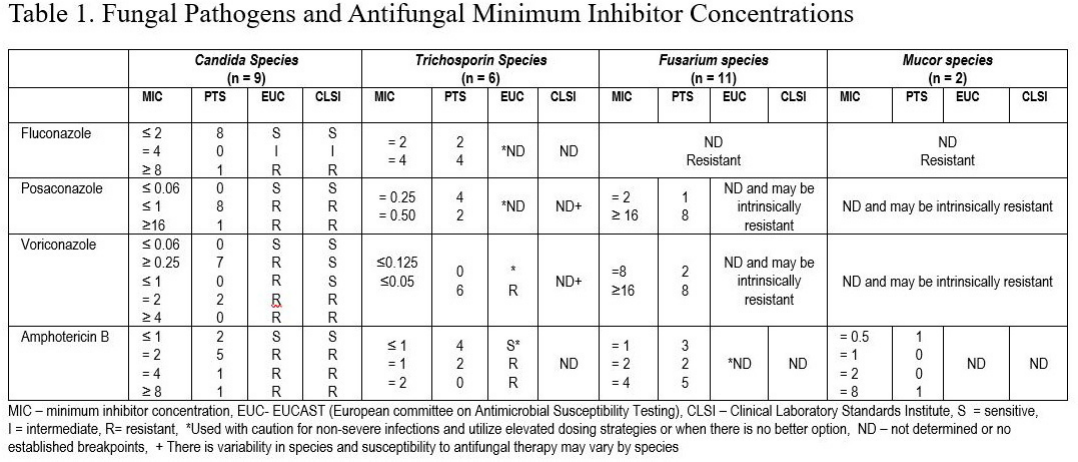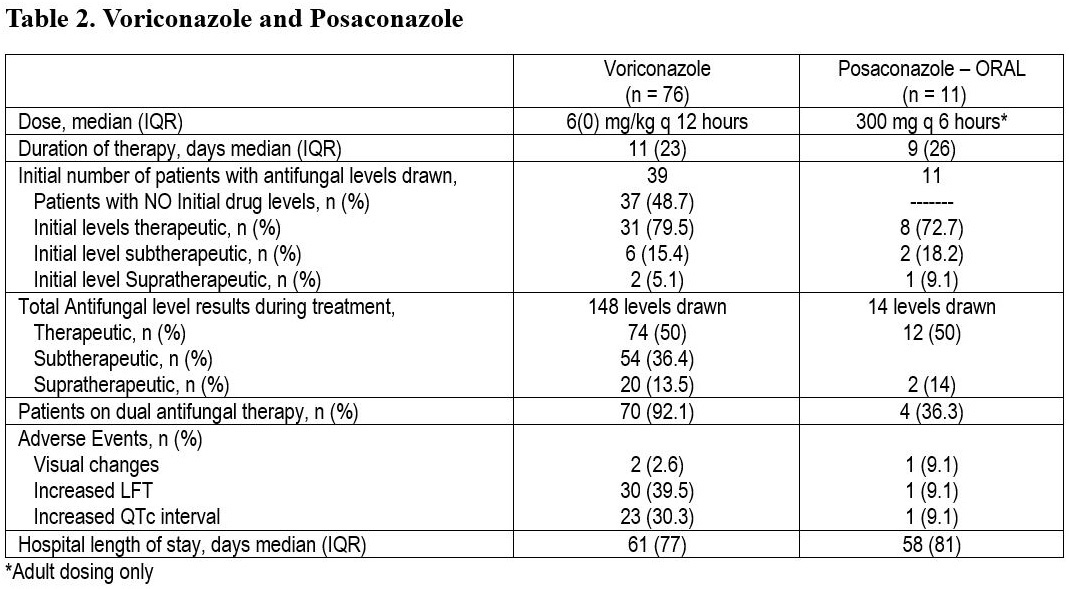# 563 Challenges of Dosing and Monitoring Antifungals in Burn Patients

**DOI:** 10.1093/jbcr/iraf019.192

**Published:** 2025-04-01

**Authors:** Janie Faris, Rebecca Coffey, Kandace Snodgrass, Courtney Rawitscher, Samuel Mandell

**Affiliations:** Parkland Health; Parkland Burn Center; Parkland Health; University of Texas Southwestern Medical School; University of Texas Southwestern / Parkland

## Abstract

**Introduction:**

Changes in drug metabolism, clearance, and volume of distribution pose unique challenges in effectively dosing antifungal agents in the burn patient. Therapeutic drug monitoring (TDM) can aid appropriate dosing despite. challenges with the timeliness of lab turnaround. This review evaluates dosing strategies, adverse events, MIC of fungal pathogens, and outcomes of fungal infections.

**Methods:**

A retrospective chart review of patients admitted to a burn center and treated with systemic antifungal agents from 2014 to 2024 was conducted. Baseline demographics, laboratory and culture data, medication administration records, drug-drug interactions, and rates of adverse events were evaluated. The primary outcomes evaluate microbiological cure, voriconazole and posaconazole levels, length of stay, and hospital mortality. Data analysis includes descriptive statistics.

**Results:**

Ninety-five patients with culture-positive fungal infections were identified. The median (IQR) TBSA was 47(40) %. Voriconazole was used in seventy-six patients, posaconazole in eleven patients (salvage therapy), and fourteen were treated with a combination of either voriconazole/posaconazole with liposomal amphotericin B. (Table 1) Of the 50 patients with TDM levels the median time to result was 8 days. Seventy-two voriconazole patients received medications that were inhibitors or substrates of CYP2C19 which may have affected voriconazole levels. Patients in the voriconazole and posaconazole group were exposed to a median (IQR) of 5(2) and 4(3) antibiotic regimens prior to antifungal therapy. (Table 2) Less than 30% of the initial fungal isolates were susceptible to echinocandins or fluconazole. Most organisms were molds (including Aspergillus species, Fusarium, Mucor/Rhizopus species, and various dematiaceous molds). Microbiological cure rate was 31(40.8%) and 6(54.5%) in the voriconazole and posaconazole regimens. Overall hospital mortality was 44%.

**Conclusions:**

The standard 4 mg/kg dose of voriconazole resulted in subtherapeutic initial levels 15% of the times. Subsequent voriconazole levels during treatment were therapeutic only 50% of the time. About half of the voriconazole patients did not have levels drawn. The levels may have been affected by the PK/PD alterations in burn patients or variability of the metabolism by CYP2C19. The turnaround time for antifungal levels delayed adjusting medication in a timely manner.

**Applicability of Research to Practice:**

More studies are needed regarding burn fungal infections, pathogens, appropriate dosing strategies and outcomes associated with MIC of the various fungal pathogens to standard therapies.

**Funding for the Study:**

N/A